# The clinical impact of estimating low-density lipoprotein cholesterol (LDL-C) using different equations in the general population

**DOI:** 10.1186/s12944-024-02188-9

**Published:** 2024-07-04

**Authors:** Reyna Lam, Sheila M. Manemann, Kristina E. Seehusen, Alan T. Remaley, Jennifer L. St. Sauver, Ruoxiang Jiang, Jill M. Killian, Maureen Sampson, Jeffrey W. Meeusen, Paul A. Decker, Véronique L. Roger, Paul Y. Takahashi, Nicholas B. Larson, Suzette J. Bielinski

**Affiliations:** 1 Department of Quantitative Health Sciences, Mayo Clinic, Rochester, MN USA; 2 University of Minnesota School of Public Health, Minneapolis, MN USA; 3 Lipoprotein Metabolism Laboratory, Translational Vascular Medicine Branch, National Heart, Lung, and Blood Institute, National Institutes of Health, Bethesda, MD USA; 4 Clinical Center, Department of Laboratory Medicine, National Institutes of Health, Bethesda, MD USA; 5 Department of Laboratory Medicine and Pathology, Mayo Clinic, Rochester, MN USA; 6 Epidemiology and Community Health Branch, National Heart, Lung, and Blood Institute, National Institutes of Health, Bethesda, MD USA; 7 Division of Community Internal Medicine, Department of Medicine, Mayo Clinic, Rochester, MN USA

**Keywords:** Low-density lipoprotein cholesterol, Friedewald, Sampson, Martin-Hopkins, Estimated low density lipoprotein cholesterol, Triglycerides

## Abstract

**Background:**

Low-density lipoprotein cholesterol (LDL-C) is associated with atherosclerotic cardiovascular disease (ASCVD). Friedewald, Sampson, and Martin-Hopkins equations are used to calculate LDL-C. This study compares the impact of switching between these equations in a large geographically defined population.

**Materials and methods:**

Data for individuals who had a lipid panel ordered clinically between 2010 and 2019 were included. Comparisons were made across groups using the two-sample t-test or chi-square test as appropriate. Discordances between LDL measures based on clinically actionable thresholds were summarized using contingency tables.

**Results:**

The cohort included 198,166 patients (mean age 54 years, 54% female). The equations perform similarly at the lower range of triglycerides but began to diverge at a triglyceride level of 125 mg/dL. However, at triglycerides of 175 mg/dL and higher, the Martin-Hopkins equation estimated higher LDL-C values than the Samson equation. This discordance was further exasperated at triglyceride values of 400 to 800 mg/dL. When comparing the Sampson and Friedewald equations, at triglycerides are below 175 mg/dL, 9% of patients were discordant at the 70 mg/dL cutpoint, whereas 42.4% were discordant when triglycerides are between 175 and 400 mg/dL. Discordance was observed at the clinically actionable LDL-C cutpoint of 190 mg/dL with the Friedewald equation estimating lower LDL-C than the other equations. In a high-risk subgroup (ASCVD risk score > 20%), 16.3% of patients were discordant at the clinical cutpoint of LDL-C < 70 mg/dL between the Sampson and Friedewald equations.

**Conclusions:**

Discordance at clinically significant LDL-C cutpoints in both the general population and high-risk subgroups were observed across the three equations. These results show that using different methods of LDL-C calculation or switching between different methods could have clinical implications for many patients.

**Supplementary Information:**

The online version contains supplementary material available at 10.1186/s12944-024-02188-9.

## Introduction

Lipoproteins, specifically low-density lipoprotein cholesterol (LDL-C), are associated with increased risk of atherosclerotic cardiovascular disease (ASCVD) [1]. As a result, a lipid panel is a commonly ordered test to help determine a patient’s risk of ASCVD. Clinical guidelines use LDL-C thresholds in combination with the presence of other ASCVD risk factors to determine therapeutic recommendations [2]. Thus, accurate estimates of LDL-C are critical as they affect the resulting clinical actions and potentially insurance coverage of guideline recommended therapies. Recommendations can range from initiation of pharmacological therapy, such as statins, for those with high LDL-C levels to lifestyle changes for those with modestly elevated levels of LDL-C.

Although direct measurements of LDL-C are more accurate, due to a variety of factors (e.g., costs, turnaround time), direct methods of measuring LDL-C levels routinely are not feasible [3, 4]. Therefore, since the 1970’s, LDL-C measurements have been calculated using the Friedewald equation [5]. The Friedewald equation uses direct measurements of total cholesterol, high-density lipoprotein cholesterol (HDL-C), and triglycerides to estimate LDL-C levels. However, it is known that these estimates can be biased, especially in the presence of high triglycerides (> 400 mg/dL). More recently, two new equations, the Sampson and Martin-Hopkins equations, have been formulated to calculate LDL-C levels to reduce the inaccuracy of LDL-C estimation [6, 7]. Studies comparing the Friedewald, Sampson, and Martin-Hopkins equations have demonstrated varying levels of accuracy. Across several studies using a variety of populations, the Sampson and Martin-Hopkins equations showed similar levels of accuracy when compared to direct measurements of LDL-C, whereas the Friedewald equation was the least accurate of the three [8–13].

Importantly, the clinical recommendations will differ for some individuals depending on the equation used to estimate LDL-C. For example, pharmacological treatment is recommended for patients with LDL-C > 190 mg/dL regardless of the presence of other risk factors. Other cutpoints, such as LDL-C greater than 100 mg/dL or 70 mg/dL, are clinically actionable depending on the presence of other risk factors such as diabetes or ASCVD 10-year risk ≥ 20% [2]. Therefore, the clinical action may differ depending on the equation used to estimate LDL-C at the time of lipid screening. Clinical labs may use different LDL-C estimating equations, which could impact the treatment for patients. For example, those with a high 10-year ASCVD risk (≥ 20%) may have varying recommended treatment regimens and/or insurance coverage due to different equations yielding conflicting LDL-C threshold results (> 70 mg/dL vs. < 70 mg/dL).

The replacement of the Friedewald equation by clinical labs with either Sampson or Martin-Hopkin equations will result in reclassification that will impact patient care around the clinical thresholds of LDL-C. Patients who may not have been recommended pharmacotherapy may now meet the requirements; conversely, those who previously met the requirements may fall below the threshold [14]. These changes have implications for patients, providers, insurers, and health care systems. However, data comparing the performance of LDL-C equations in the general population are lacking. Therefore, in this study, we focused on a large, geographically defined population and used lipid measurements ordered for routine clinical care to estimate LDL-C using the three equations for each patient. The LDL-C levels were compared by equation, and we summarize the impact of each patient’s LDL-C per equation on clinical recommendations.

## Materials and methods

### Data source

The Rochester Epidemiology Project (REP) is a system linking medical records [15]. The REP includes electronic health record (EHR) data for persons who have lived in a 27-county region in Southeastern Minnesota and Southwestern Wisconsin after January 1, 2010 [16, 17]. The REP includes EHR from Mayo Clinic, Mayo Clinic Health System clinics and hospitals, and Olmsted Medical Center and its affiliated clinics. The REP captures approximately 61% of the entire population residing in this region [16]. For this study, we used nine of the 27 counties within the REP capture area and include Dodge, Fillmore, Freeborn, Goodhue, Mower, Olmsted, Steele, Wabasha, and Waseca counties. These nine counties were selected due to the large percentage (> 90%) of the population captured by the REP [17].

### Study population

Using the REP, individuals ages 30 and older with Minnesota Research Authorization and residency in the nine-county region who had a lipid panel ordered for clinical screening in the years 2010 to 2019 were included. The index date was the date of the first lipid panel with measurements of total cholesterol, HDL-C, and triglycerides within the data collection period. We did not require an LDL-C estimate report given the inconsistencies of reporting LDL-C at high triglyceride levels. Furthermore, we excluded those with a triglyceride level of ≥ 800 mg/dL (*n* = 539) as LDL-C estimation is not reliable above this threshold for any of the three equations.

### LDL-C estimation and clinical predictors

LDL-C was estimated by each equation and subsequently referred to as the Friedewald, Sampson, and Martin-Hopkins LDL-C [5–7]. The values of all demographic and clinical variables were obtained from the EHR and included age, sex, race, and ethnicity of patients at the index date. Race and ethnicity were classified per United States Census criteria. Body mass index (BMI; kg/m^2^), smoking status, systolic blood pressure (mm Hg), and diastolic blood pressure (mm Hg) were also retrieved. Diabetes was determined by the presence of a diagnosis code. All clinical variables were obtained as close to the index date as possible within a five-year lookback window. Patients who received a prescription for an anti-hypertensive therapy in the two years prior to index were considered on hypertension therapy. The ASCVD pooled risk equation was used to estimate the 10-year risk of an ASCVD event for each patient [18]. Any missing quantitative values were assigned the midpoint of the normal range and the low-risk value for dichotomous values (e.g., non-smoker).

### Data analyses

Patient characteristics were summarized using mean (standard deviation), select percentiles, and number (percent) as appropriate. Summaries were done overall and by lipid lowering therapy. Comparisons were made across groups using the two-sample t-test or chi-square test as appropriate. Discordances between LDL measures based on clinically actionable thresholds were summarized using contingency tables.

## Results

The characteristics of the study population are provided in Table 1 for the entire cohort and stratified by use of lipid-lowering therapy. The cohort included 198,166 patients. The mean age of the cohort was 54 years and 54% were female. The study population was predominately white race; however, there were 5,122 Black, 5,244 Asian, and 5,041 other or mixed race. In addition, 8,622 were Hispanic. When compared to those who were not using lipid-lowering therapy, those who used lipid-lowering therapy were older, more likely to be male, white, and non-Hispanic. Those taking lipid-lowering therapies were also more likely to be diabetic, using hypertension therapy, and had lower cholesterol levels. The distribution of triglycerides in the study population is illustrated (See Supplementary Fig. 1, Additional File 1). In addition, a graph visualizing non-HDL (mg/dL) levels by triglyceride (mg/dL) levels from 0 to 800 is shown (See Supplementary Fig. 2, Additional File 1).

**Table 1:** Characteristics of the study population, count (percentage) or mean (standard deviation)

Characteristics	Full study population	Lipid lowering therapy		
		No	Yes	*P* value
*n*	198,166	151,669	46,497	
Age, years	54 (15)	51 (14)	64 (13)	< 0.001
Age categories, years				< 0.001
30–39	39,177 (20)	37,433 (25)	1744 (3.8)	
40–49	41,467 (21)	36,866 (24)	4601 (9.9)	
50–59	48,557 (25)	37,871 (25)	10,686 (23)	
60–69	34,253 (17)	21,821 (14)	12,432 (27)	
70–79	20,992 (11)	10,786 (7.1)	10,206 (22)	
80+	13,720 (6.9)	6892 (4.5)	6828 (15)	
Sex, female	106,474 (54)	84,869 (56)	21,605 (47)	< 0.001
Race				< 0.001
Black	5122 (2.6)	4461 (2.9)	661 (1.4)	
Asian	5244 (2.6)	4436 (2.9)	808 (1.7)	
Hawaiian/Pacific Islander	254 (0.1)	206 (0.1)	48 (0.1)	
American Indian	655 (0.3)	520 (0.3)	135 (0.3)	
Other/Mixed	5041 (2.5)	4135 (2.7)	906 (1.9)	
White	180,327 (91)	136,616 (90)	43,711 (94)	
Unknown	1523 (0.8)	1295 (0.9)	228 (0.5)	
Hispanic ethnicity	8622 (4.4)	7208 (4.8)	1414 (3.0)	
BMI (kg/m^2^)	30.0 (6.8)	29.7 (6.9)	31 (6.4)	< 0.001
Missing	33,237	28,569	4668	
Smoking status				< 0.001
Current	26,311 (13)	20,757 (14)	5554 (12)	
Former	41,112 (21)	29,087 (19)	12,025 (26)	
Never	97,921 (49)	74,267 (49)	23,654 (51)	
Unknown	32,822 (17)	27,558 (18)	5264 (11)	
Systolic Blood Pressure, mm Hg	123 (17)	122 (17)	126 (17)	< 0.001
Missing	21,221	19,474	1747	
Diastolic Blood Pressure, mm Hg	75 (11)	75 (11)	72 (11)	< 0.001
Missing	21,221	19,474	1747	
Use of Hypertension Therapy	57,563 (29)	28,007 (19)	29,556 (64)	< 0.001
Diabetic	45,982 (23)	23,233 (15)	22,749 (49)	< 0.001
Total Cholesterol, mg/dL	192 (40)	196 (39)	179 (42)	< 0.001
Range	40–748	42–748	40–585	
HDL Cholesterol, mg/dL	53 (17)	54 (18)	49 (15)	< 0.001
Range	3–249	3–249	3–172	
Triglycerides, mg/dL	137 (85)	132 (82)	154 (89)	< 0.001
Range	8–799	8–799	19–797	
LDL Cholesterol, mg/dL-Friedewald	112 (35)	116 (34)	99 (36)	< 0.001
Range	0–614	0–614	0–487	
LDL Cholesterol, mg/dL-Martin	115 (340)	118 (34)	104(35)	< 0.001
Range	0–636	0–636	8.5–492	
LDL Cholesterol, mg/dL-Sampson	115 (35)	118 (34)	103 (35)	< 0.001
Range	0–528	0–528	8.4–470	
ASCVD Risk	9.5 (11)	7.2 (9.2)	16 (14)	< 0.001
Sample size	145,269	107,344	37,925	
*Participants with triglycerides < 400 mg/dL*	**194,720**	**149,289**	**45,431**	
Total Cholesterol, mg/dL	191(40)	195 (38)	178 (41)	< 0.001
Range	40–705	42–705	40–585	
LDL Cholesterol, mg/dL-Friedewald	112 (35)	116 (34)	100 (35)	< 0.001
Range	0–566	0–566	0–487	
LDL Cholesterol, mg/dL-Martin	115 (34)	118 (33)	104 (35)	< 0.001
Range	0–578	0–578	8.5–492	
LDL Cholesterol, mg/dL-Sampson	115 (35)	119 (34)	103 (35)	< 0.001
Range	0–521	0–521	8.4–470	
*Participants with triglycerides ≥ 400 mg/dL*	**3446**	**2380**	**1066**	
Total Cholesterol, mg/dL	234 (53)	236 (51)	229 (56)	< 0.001
Range	102–748	108–748	102–542	
LDL Cholesterol, mg/dL-Friedewald	96 (50)	99 (49)	91 (51)	< 0.001
Range	0–614	0–614	0–351	
LDL Cholesterol, mg/dL-Martin	128 (41)	130 (40)	124 (42)	< 0.001
Range	28–636	26–636	33–376	
LDL Cholesterol, mg/dL-Sampson	110 (40)	112 (39)	106 (42)	< 0.001
Range	6.7–528	6.7–528	15–309	

*BMI* body mass index, *HDL* high-density lipoprotein, *LDL* lipoprotein, *ASCVD* atherosclerotic cardiovascular disease

**Fig. 1:**
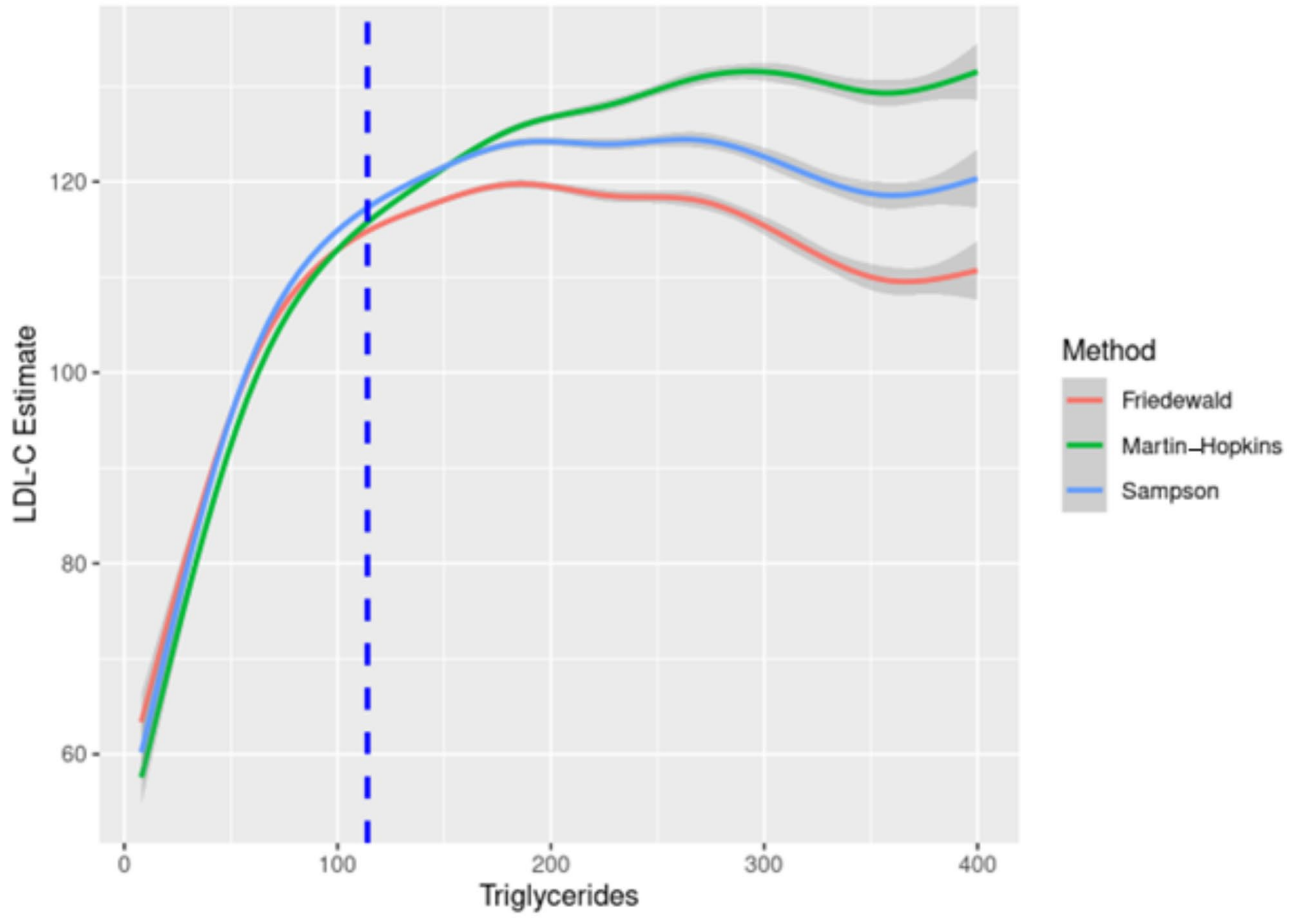
Estimated low-density lipoprotein cholesterol (LDL-C) by triglyceride levels for each equation. The median triglyceride level is represented by the dotted blue line

Figure 1 compares the mean estimated LDL-C by equation for patients with triglycerides < 400 mg/dL. The three equations perform similarly in estimating LDL-C at the lower range of triglycerides. However, beginning at a triglyceride level of 125 mg/dL, estimates of LDL-C via Friedewald equation begin to deviate from Sampson and Martin-Hopkins. Likewise, beginning around a triglyceride level of 175 mg/dL, the Sampson and Martin-Hopkins equations begin to diverge. Due to the limitations of the Friedewald equation, for triglycerides 400 mg/dL to 800 mg/dL, only the Sampson and Martin-Hopkins equations are compared. Across this range of triglyceride values, the Sampson equation consistently estimates lower LDL-C values in comparison to the Martin-Hopkins equation (See Supplementary Fig. 3, Additional File 1).

A comparison of the Sampson and Friedewald LDL-C estimates for those with triglycerides ≤ 400 mg/dL and stratified by triglycerides levels of < 175 mg/dL and 175–400 mg/dL are provided in Table 2 by relevant clinical cutpoints. Overall, the discordance between the Sampson and Friedewald equations is greatest at low LDL-C values and gradually declines as LDL-C values increase. However, the discordance is most pronounced at triglyceride levels between 175 and 400 mg/dL. When triglycerides are below 175 mg/dL, 1434/15,994 (9%: 95% CI: 8.5 − 9.4%) of patients were discordant at the 70 mg/dL cutpoint, whereas 1763/4162 (42%; 95% CI: 40.9 − 43.9%) were discordant when triglycerides are between 175 and 400 mg/dL. Similarly, we compared the Martin-Hopkins, Friedewald, and the Sampson equations. The pattern of discordance between Martin-Hopkins and Friedewald is similar to comparisons of the Sampson and Friedewald equations, albeit the discordance was greater (See Supplementary Table 1, Additional File 1). The Sampson and Martin-Hopkins equations show less discordance; however differences at the clinic cutpoints are still present and are greater at higher triglyceride levels (See Supplementary Table 2, Additional File 1).

**Table 2:** Comparison of LDL-C estimated by the Friedewald and Sampson equations for those participants with triglycerides < 400 mg/dL and further stratified by triglycerides < 175 and 175 < 400 mg/dL, count (%)

**Triglycerides < 400 mg/dL**		**Friedewald**						
	**LDL-C Value (mg/dL)**		Desirable ≤ 70 (*N* = 20,156)	Desirable 71–99 (*N* = 54,726)	Above Desirable 100–129 (*N* = 64,937)	Borderline High 130–159 (*N* = 37,925)	High 160–189 (*N* = 12,936)	Very High ≥ 190 (*N* = 4040)
**Sampson**	Desirable ≤ 70		16,959 (84.1)	251 (0.5)	0 (0.0)	0 (0.0)	0 (0.0)	0 (0.0)
	Desirable 71–99		3197 (15.9)	48,204 (88.1)	29 (0.0)	0 (0.0)	0 (0.0)	0 (0.0)
	Above Desirable 100–129		0 (0.0)	6271 (11.5)	59,497 (91.6)	0 (0.0)	0 (0.0)	0 (0.0)
	Borderline High 130–159		0 (0.0)	0 (0.0)	5411 (8.3)	35,677 (94.1)	0 (0.0)	0 (0.0)
	High 160–189		0 (0.0)	0 (0.0)	0 (0.0)	2248 (5.9)	12,466 (96.4)	1 (0.0)
	Very High ≥ 190		0 (0.0)	0 (0.0)	0 (0.0)	0 (0.0)	470 (3.6)	4039 (100.0)
**Triglycerides < 175 mg/dL**			**Friedewald**					
	**LDL-C Value (mg/dL)**		Desirable ≤ 70 (*N* = 15,994)	Desirable 71–99 (*N* = 44,869)	Above Desirable 100–129 (*N* = 51,962)	Borderline High 130–159 (*N* = 28,828)	High 160–189 (*N* = 8994)	Very High ≥ 190 (*N* = 2440)
**Sampson**	Desirable ≤ 70		14,560 (91.0)	251 (0.6)	0 (0.0)	0 (0.0)	0 (0.0)	0 (0.0)
	Desirable 71–99		1434 (9.0)	41,115 (91.6)	29 (0.1)	0 (0.0)	0 (0.0)	0 (0.0)
	Above Desirable 100–129		0 (0.0)	3503 (7.8)	48,592 (93.5)	0 (0.0)	0 (0.0)	0 (0.0)
	Borderline High 130–159		0 (0.0)	0 (0.0)	3341 (6.4)	27,345 (94.9)	0 (0.0)	0 (0.0)
	High 160–189		0 (0.0)	0 (0.0)	0 (0.0)	1483 (5.1)	8646 (96.1)	0 (0.0)
	Very High ≥ 190		0 (0.0)	0 (0.0)	0 (0.0)	0 (0.0)	348 (3.9)	2440 (100.0)
**Triglycerides 175 < 400 mg/dL**			**Friedewald**					
	**LDL-C Value (mg/dL)**		Desirable ≤ 70 (*N* = 4162)	Desirable 71–99 (*N* = 9857)	Above Desirable 100–129 (*N* = 12,975)	Borderline High 130–159 (*N* = 9097)	High 160–189 (*N* = 3942)	Very High ≥ 190 (*N* = 1600)
**Sampson**	Desirable ≤ 70		2399 (57.6)	0 (0.0)	0 (0.0)	0 (0.0)	0 (0.0)	0 (0.0)
	Desirable 71–99		1763 (42.4)	7089 (71.9)	0 (0.0)	0 (0.0)	0 (0.0)	0 (0.0)
	Above Desirable 100–129		0 (0.0)	2768 (28.1)	10,905 (84.0)	0 (0.0)	0 (0.0)	0 (0.0)
	Borderline High 130–159		0 (0.0)	0 (0.0)	2070 (16.0)	8332 (91.6)	0 (0.0)	0 (0.0)
	High 160–189		0 (0.0)	0 (0.0)	0 (0.0)	765 (8.4)	3820 (96.9)	1 (0.1)
	Very High ≥ 190		0 (0.0)	0 (0.0)	0 (0.0)	0 (0.0)	122 (3.1)	1599 (99.9)

*LDL-C* lipoprotein cholesterol

**Fig. 2:**
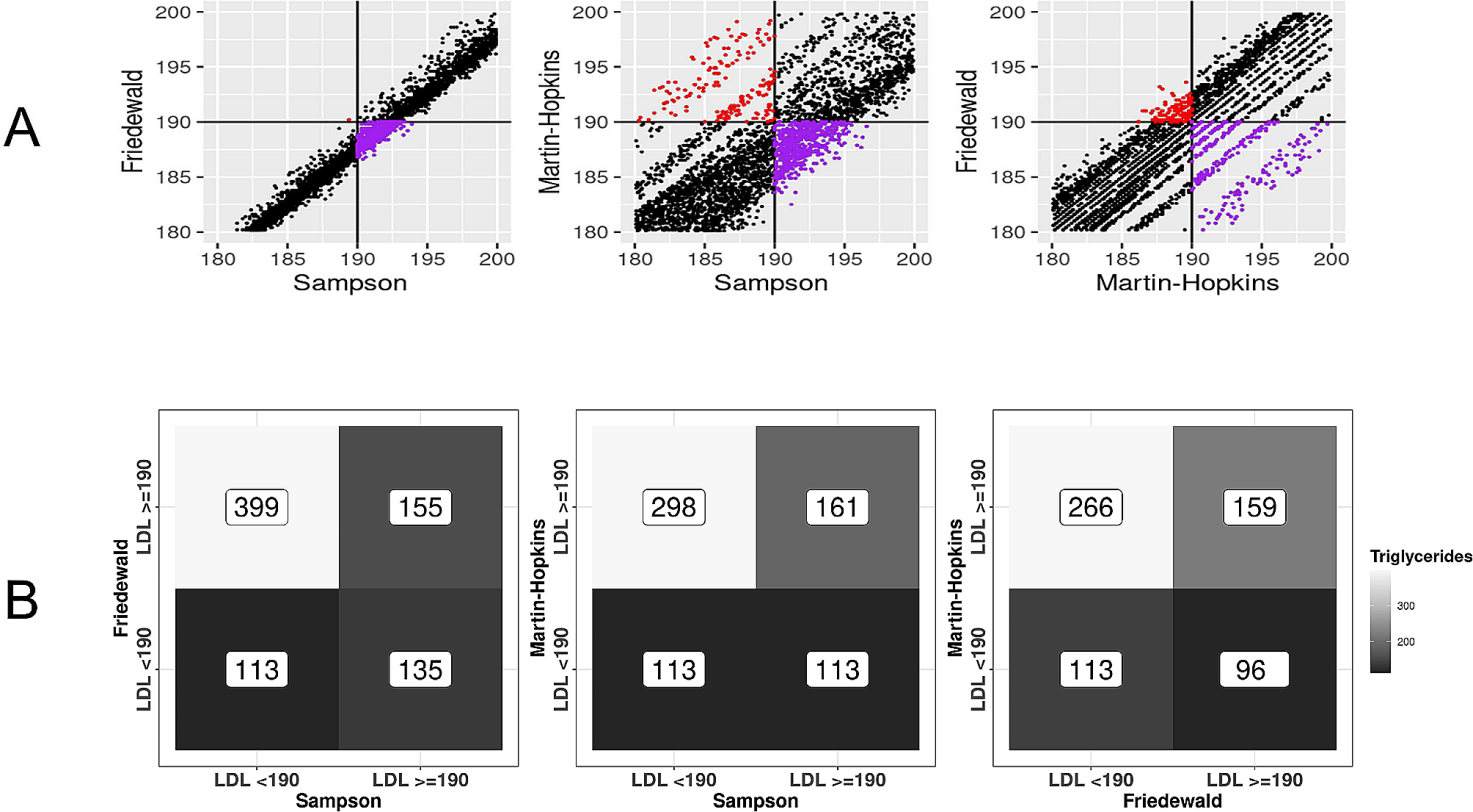
Comparison of high low-density lipoprotein cholesterol (LDL-C) estimated by equation for those patients with triglycerides < 400 mg/dL. **(A)** Concordance and discordance around LDL values of 190 mg/dL and **(B)** median triglyceride level for each quadrant

**Fig. 3:**
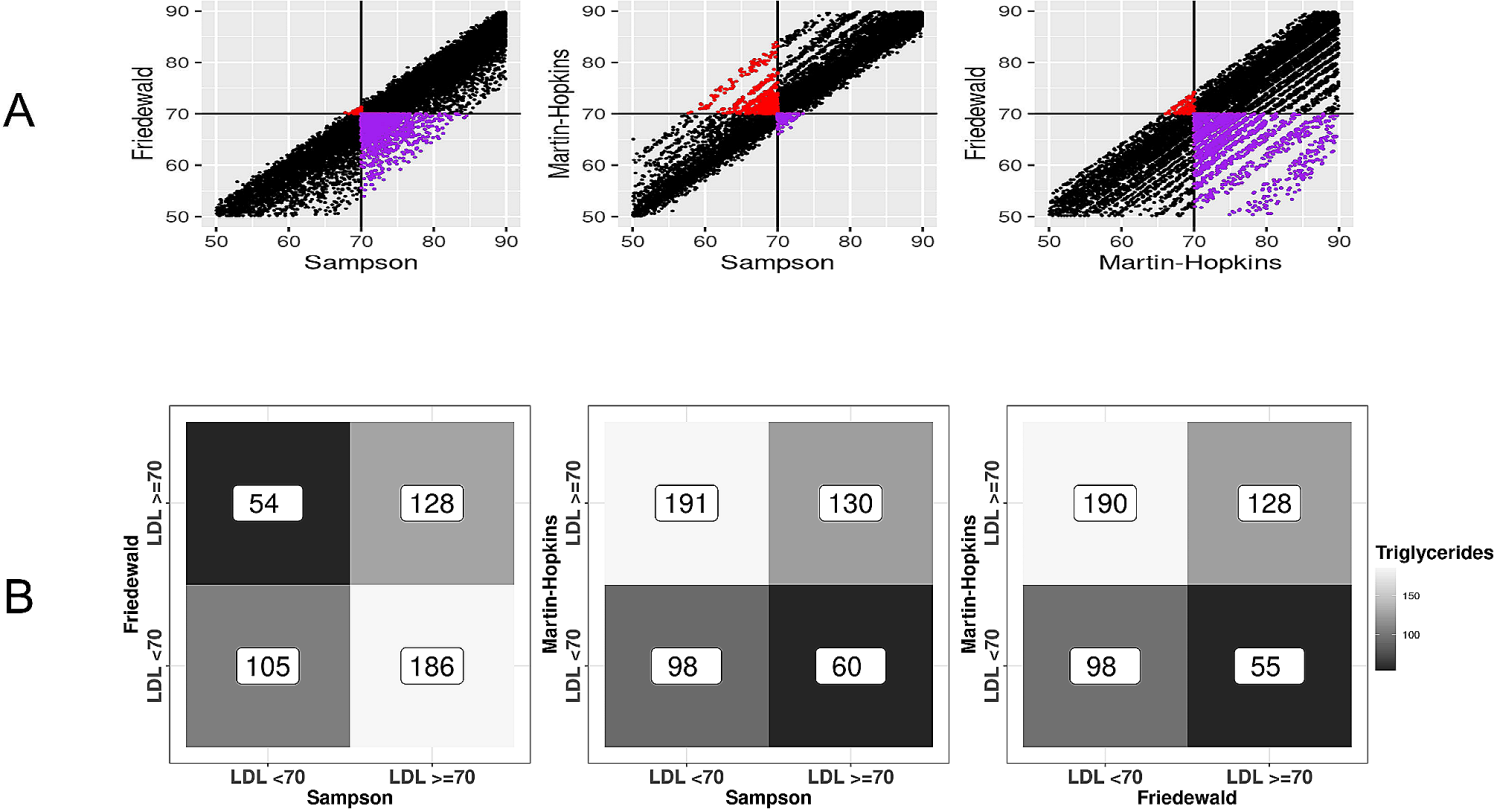
Comparison of low-density lipoprotein cholesterol (LDL-C) for those patients with triglycerides < 400 mg/dL and high 10-year atherosclerotic cardiovascular disease risk (ASCVD ≥ 20%). **(A)** Concordance and discordance around LDL values of 70 mg/dL and **(B)** median triglyceride level for each quadrant

Figure 2 illustrates the two-by-two comparisons focusing on the clinically actionable LDL-C cutpoint of 190 mg/dL. Discordance around this cutpoint was observed for each pair compared. The Friedewald and Sampson comparison, as well as the Friedewald and Martin-Hopkins comparison show a similar tendency of the Friedewald equation to produce lower LDL-C estimates than the two other equations. Table 3 compares the Friedewald and Sampson equations at clinical LDL-C cutpoints when triglycerides are below 400 mg/dL stratified by 10-year ASCVD risk. The discordance pattern of the Friedewald equation estimating lower LDL-C values than the Sampson equation was similar between the Friedewald and Sampson equations across ASCVD risk ranges. Classifications from the two scores converge for LDL values ≥ 190 mg/dL such that either the Sampson or the Friedwald score would classify a patient as “very high” risk. However, regardless of ASCVD risk, the Sampson equation consistently classified more patients in higher categories compared to the Friedwald score. For example, among patients with an ASCVD risk ≥ 20%, 6.3% (95% CI: 5.6 − 7.0%) of patients who were classified as “borderline high” (130–159) were re-classified as “high” when the Sampson equation was used instead of the Friedwald equation. See Supplementary Table 3, Additional File 1, which shows this comparison for Sampson and Martin-Hopkins. Figure 3 illustrates the two-by-two comparisons for this high-risk subgroup. Similarly, we compared the equations for patients with diabetes and a 10-year ASCVD risk of ≥ 7.5% around the LDL-C cutpoint of 70 mg/dL (See Supplementary Fig. 4, Additional File 1) and a low ASCVD risk around the LDL-C cutpoint of 100 mg/dL (See Supplementary Fig. 5, Additional File 1). For all clinically relevant comparisons, we observed similar levels of discordance by equation. Further visualizations of the discordance between all three equations are shown through rose plots (See Supplementary Fig. 6, Additional File 1).

**Table 3:** Comparison of LDL-C estimated by the Friedewald and Sampson equations stratified by 10-Year ASCVD risk for those participants with triglycerides < 400 mg/dL, count (%)

**ASCVD risk < 7.5%**		**Friedewald**					
	**LDL Value (mg/dL)**	Desirable ≤ 70 (*N* = 10,077)	Desirable 71–99 (*N* = 33,993)	Above Desirable 100–129 (*N* = 44,397)	Borderline High 130–159 (*N* = 25,713)	High 160–189 (*N* = 8192)	Very High ≥ 190 (*N* = 2261)
**Sampson**	Desirable ≤ 70	8625 (85.6)	191 (0.6)	0 (0.0)	0 (0.0)	0 (0.0)	0 (0.0)
	Desirable 71–99	1452 (14.4)	30,097 (88.5)	23 (0.1)	0 (0.0)	0 (0.0)	0 (0.0)
	Above Desirable 100–129	0 (0.0)	3705 (10.9)	40,751 (91.8)	0 (0.0)	0 (0.0)	0 (0.0)
	Borderline High 130–159	0 (0.0)	0 (0.0)	3623 (8.2)	24,204 (94.1)	0 (0.0)	0 (0.0)
	High 160–189	0 (0.0)	0 (0.0)	0 (0.0)	1509 (5.9)	7906 (96.5)	1 (0.0)
	Very High ≥ 190	0 (0.0)	0 (0.0)	0 (0.0)	0 (0.0)	286 (3.5)	2260 (100.0)
**ASCVD risk 7.5 < 20%**		**Friedewald**					
	**LDL Value (mg/dL)**	Desirable ≤ 70 (*N* = 3451)	Desirable 71–99 (*N* = 9247)	Above Desirable 100–129 (*N* = 11,589)	Borderline High 130–159 (*N* = 7690)	High 160–189 (*N* = 3181)	Very High ≥ 190 (*N* = 1184)
**Sampson**	Desirable ≤ 70	2787 (80.8)	14 (0.2)	0 (0.0)	0 (0.0)	0 (0.0)	0 (0.0)
	Desirable 71–99	664 (19.2)	7964 (86.1)	1 (0.0)	0 (0.0)	0 (0.0)	0 (0.0)
	Above Desirable 100–129	0 (0.0)	1269 (13.7)	10,537 (90.9)	0 (0.0)	0 (0.0)	0 (0.0)
	Borderline High 130–159	0 (0.0)	0 (0.0)	1051 (9.1)	7237 (94.1)	0 (0.0)	0 (0.0)
	High 160–189	0 (0.0)	0 (0.0)	0 (0.0)	453 (5.9)	3050 (95.9)	0 (0.0)
	Very High ≥ 190	0 (0.0)	0 (0.0)	0 (0.0)	0 (0.0)	131 (4.1)	1184 (100.0)
**ASCVD risk ≥ 20%**		**Friedewald**					
	**LDL Value (mg/dL)**	Desirable ≤ 70 (*N* = 6628)	Desirable 71–99 (*N* = 11,486)	Above Desirable 100–129 (*N* = 8951)	Borderline High 130–159 (*N* = 4522)	High 160–189 (*N* = 1563)	Very High ≥ 190 (*N* = 595)
**Sampson**	Desirable ≤ 70	5547 (83.7)	46 (0.4)	0 (0.0)	0 (0.0)	0 (0.0)	0 (0.0)
	Desirable 71–99	1081 (16.3)	10,143 (88.3)	5 (0.1)	0 (0.0)	0 (0.0)	0 (0.0)
	Above Desirable 100–129	0 (0.0)	1297 (11.3)	8209 (91.7)	0 (0.0)	0 (0.0)	0 (0.0)
	Borderline High 130–159	0 (0.0)	0 (0.0)	737 (8.2)	4236 (93.7)	0 (0.0)	0 (0.0)
	High 160–189	0 (0.0)	0 (0.0)	0 (0.0)	286 (6.3)	1510 (96.6)	0 (0.0)
	Very High ≥ 190	0 (0.0)	0 (0.0)	0 (0.0)	0 (0.0)	53 (3.4)	595 (100.0)

*LDL-C* lipoprotein cholesterol, *ASCVD* atherosclerotic cardiovascular disease

## Discussion

In this study, we examined the potential clinical impact of using different methods of LDL-C estimation in a large geographically defined population. Specifically, we compared the newer Sampson and Martin-Hopkins equations to the Friedewald equation. Of the 198,166 patients in the cohort, we focused on specific subsets of patients that would be most significantly affected by a change in methodology of LDL-C estimation. We observed discordance at clinically significant cutpoints of LDL-C, both in the general population as well as in high-risk subgroups. These results demonstrate that the use of different equations and or switching between equations will have clinical implications for a substantial number of patients.

Comparisons between the Friedewald, Sampson, and Martin-Hopkins have been previously explored [6–13, 19–21]. However, prior research has not compared these three equations in a cohort this large and diverse. Our findings showed patterns consistent with prior studies highlighting the tendency of the Friedewald equation to estimate lower LDL-C values than the Sampson and Martin-Hopkins equations. In addition, we also found LDL-C discordance to be dependent on triglyceride levels with discordance increasing as triglycerides increase [6, 7, 21].

Our results are consistent with previous studies showing that the Sampson and Martin-Hopkins equations estimate higher LDL-C values compared to the Friedewald equation, especially when triglyceride values are greater than 400 mg/dL [7, 11, 19, 21]. Previous studies have shown that the Sampson and Martin-Hopkins equations are more accurate in comparison to the Friedewald Eq. [7]. Therefore, use of these two new equations may be beneficial in a clinical setting allowing more patients to be eligible for treatments and interventions to reduce LDL-C, particularly in patients where LDL values are < 190 mg/dL. Our results indicate that all three equations converge when LDL values are very high (≥ 190 mg/dL), such that patients with such LDL values will be classified as needing therapy regardless of which equation is used. However, as compared to Friedewald, use of the Sampson equation will reclassify patients from borderline high or high LDL categories into high or very high LDL categories, regardless of ASCVD risk score. Thus, the Sampson equation may substantially increase the number of patients eligible for statin therapy.

The strengths of our study include a large geographically defined real-world population undergoing lipid screening. Furthermore, we focused on three LDL-C estimating equations currently in use in clinical laboratories and on key subsets of the population where discordance in LDL-C would impact clinical care and insurance coverage. Our study has some limitations of note. First, we used routine clinical lipid panel data and not direct measures of LDL-C, therefore we did not have a gold-standard measurement to compare to each equation’s estimate. Rather, each equation was compared to the others using discordance as a means of describing the potential implications for clinical care. In addition, the study used lipid panel results ordered for a patient’s clinical care. Patients are instructed to fast prior to the blood draw if triglycerides are measured but fasting is self-reported and thus may not be completely accurate. Previous studies have shown that in the non-fasting state, triglyceride levels can increase while LDL-C and HDL-C levels can decrease in comparison to a fasting state [21].

## Conclusion

While previous studies have compared the Friedewald, Sampson, and Martin-Hopkins equations, these studies have not explored potential ramifications of equation use in the general population. We observed discordance at clinically significant cutpoints of LDL-C, both in the general population as well as in high-risk subgroups. These results demonstrate that the use of different equations and or switching between equations will have clinical implications for a substantial number of patients. Based on these findings, clinical laboratories should indicate the equation or method used to measure LDL-C.

### Electronic supplementary material

Below is the link to the electronic supplementary material.

Additional file 1: **Table S1.** Comparison of LDL-C estimated by the Friedewald and Martin-Hopkins Equations for those participants with triglycerides < 400 mg/dL and further stratified by triglycerides < 175 and 175 < 400 mg/dL, count (%). **Table S2.** Comparison by LDL-C estimated by the Martin-Hopkins and Sampson Equations stratified by triglycerides levels, count (%). **Table S3.** Comparison of LDL-C estimated by the Sampson and Martin-Hopkins Equations stratified by 10-Year ASCVD Risk for those participants with triglycerides < 400 mg/dL, count (%). **Fig. S1.** Distribution of triglycerides in the study population. The median triglyceride level is represented by the dotted blue line. **Fig. S2.** Non-HDL (mg/dL) levels by triglyceride levels for triglyceride 0-800 mg/dL. **Fig. S3.** Estimated low-density lipoprotein cholesterol (LDL-C) by triglyceride levels for Martin-Hopkins and Sampson when triglyceride (400 mg/dL – 800 mg/dL). **Fig. S4.** Comparison of low-density lipoprotein cholesterol (LDL-C) for diabetic patients with triglycerides < 400 mg/dL and 10-year atherosclerotic cardiovascular disease risk (ASCVD) ≥ 7.5%. (A) Concordance and discordance around LDL values of 70 mg/dL and (B) median triglyceride level for each quadrant. **Fig. S5.** Comparison of low-density lipoprotein cholesterol (LDL-C) for diabetic patients with triglycerides < 400 mg/dL and low 10-year atherosclerotic cardiovascular disease risk (ASCVD) < 7.5%. (A) Concordance and discordance around LDL values of 100 mg/dL and (B) median triglyceride level for each quadrant. **Fig. S6.** Comparison of low-density lipoprotein cholesterol (LDL-C) at the 190 mg/dL threshold by equation.

## Data Availability

The data from this study are available from the corresponding author on reasonable request.

## Abbreviations

ASCVD: Atherosclerotic cardiovascular disease
BMI: Body mass index
EHR: Electronic health record
HDL-C: High-density lipoprotein cholesterol
LDL-C: Low-density lipoprotein cholesterol
REP: Rochester Epidemiology Project
