# Supplementary material for: The clinical impact of estimating low-density lipoprotein cholesterol (LDL-C) using different equations in the general population
Source: Lipids Health Dis. 2024 Jul 4;23:210. doi: 10.1186/s12944-024-02188-9 (PMC11223329; doi:10.1186/s12944-024-02188-9)

Supplementary Material - Additional File 1

The clinical impact of estimating low-density lipoprotein cholesterol (LDL-C) using different equations in the general population

Reyna Lam, Sheila M. Manemann, Kristina E. Seehusen, Alan T. Remaley, Jennifer L. St. Sauver, Ruoxiang Jiang, Jill M. Killian, Maureen Sampson, Jeffrey W. Meeusen, Paul A. Decker, Véronique L. Roger, Paul Y. Takahashi, Nicholas B. Larson, and Suzette J. Bielinski

Supplementary Table 1 Comparison of LDL-C estimated by the Friedewald and Martin-Hopkins Equations for those participants with triglycerides < 400 mg/dL and further stratified by triglycerides <175 and 175<400 mg/dL, count (%)

Supplementary Table 2 Comparison by LDL-C estimated by the Martin-Hopkins and Sampson Equations stratified by triglycerides levels, count (%)

Supplementary Table 3 Comparison of LDL-C estimated by the Sampson and Martin-Hopkins Equations stratified by 10-Year ASCVD Risk for those participants with triglycerides < 400 mg/dL, count (%)

Supplementary Fig. 1 Distribution of triglycerides in the study population. The median triglyceride level is represented by the dotted blue line.

Supplementary Fig. 2 Non-HDL (mg/dL) levels by triglyceride levels for triglyceride 0-800 mg/dL.

Supplementary Fig. 3 Estimated low-density lipoprotein cholesterol (LDL-C) by triglyceride levels for Martin-Hopkins and Sampson when triglyceride (400 mg/dL – 800 mg/dL)

Supplementary Fig. 4 Comparison of low-density lipoprotein cholesterol (LDL-C) for diabetic patients with triglycerides < 400 mg/dL and 10-year atherosclerotic cardiovascular disease risk

(ASCVD) ≥ 7.5%. (A) Concordance and discordance around LDL values of 70 mg/dL and (B) median triglyceride level for each quadrant.

Supplementary Fig. 5 Comparison of low-density lipoprotein cholesterol (LDL-C) for diabetic patients with triglycerides < 400 mg/dL and low 10-year atherosclerotic cardiovascular disease risk (ASCVD) < 7.5%. (A) Concordance and discordance around LDL values of 100 mg/dL and (B) median triglyceride level for each quadrant.

Supplementary Fig. 6 Comparison of low-density lipoprotein cholesterol (LDL-C) at the 190 mg/dL threshold by equation.

Supplementary Table 1 Comparison of LDL-C estimated by the Friedewald and Martin-Hopkins Equations for those participants with triglycerides < 400 mg/dL and further stratified by triglycerides <175 and 175<400 mg/dL, count (%)

**Triglycerides <400 mg/dL**		**Friedewald**					
	**LDL Value (mg/dL)**	Desirable≤70 (N=20156)	Desirable 71-99 (N=54726)	Above Desirable 100-129 (N=64937)	Borderline High 130-159 (N=37925)	High 160-189 (N=12936)	Very High≥190 (N=4040)
**Martin-Hopkins**	Desirable≤70	15620 (77.5%)	865 (1.6%)	0 (0.0%)	0 (0.0%)	0 (0.0%)	0 (0.0%)
	Desirable 71-99	4536 (22.5%)	46398 (84.8%)	1690 (2.6%)	0 (0.0%)	0 (0.0%)	0 (0.0%)
	Above Desirable 100-129	0 (0.0%)	7463 (13.6%)	57812 (89.0%)	1232 (3.2%)	0 (0.0%)	0 (0.0%)
	Borderline High 130-159	0 (0.0%)	0 (0.0%)	5435 (8.4%)	34823 (91.8%)	540 (4.2%)	0 (0.0%)
	High 160-189	0 (0.0%)	0 (0.0%)	0 (0.0%)	1870 (4.9%)	12121 (93.7%)	140 (3.5%)
	Very High≥190	0 (0.0%)	0 (0.0%)	0 (0.0%)	0 (0.0%)	275 (2.1%)	3900 (96.5%)
**Triglycerides <175 mg/dL**		**Friedewald**					
	**LDL Value (mg/dL)**	Desirable≤70 (N=15994)	Desirable 71-99 (N=44869)	Above Desirable 100-129 (N=51962)	Borderline High 130-159 (N=28828)	High 160-189 (N=8994)	Very High≥190 (N=2440)
**Martin-Hopkins**	Desirable≤70	14194 (88.7%)	865 (1.9%)	0 (0.0%)	0 (0.0%)	0 (0.0%)	0 (0.0%)
	Desirable 71-99	1800 (11.3%)	41282 (92.0%)	1690 (3.3%)	0 (0.0%)	0 (0.0%)	0 (0.0%)
	Above Desirable 100-129	0 (0.0%)	2722 (6.1%)	48672 (93.7%)	1232 (4.3%)	0 (0.0%)	0 (0.0%)
	Borderline High 130-159	0 (0.0%)	0 (0.0%)	1600 (3.1%)	27301 (94.7%)	540 (6.0%)	0 (0.0%)
	High 160-189	0 (0.0%)	0 (0.0%)	0 (0.0%)	295 (1.0%)	8452 (94.0%)	140 (5.7%)
	Very High≥190	0 (0.0%)	0 (0.0%)	0 (0.0%)	0 (0.0%)	2 (0.0%)	2300 (94.3%)
**Triglycerides 175<400 mg/dL**		**Friedewald**					
	**LDL Value (mg/dL)**	Desirable≤70 (N=4162)	Desirable 71-99 (N=9857)	Above Desirable 100-129 (N=12975)	Borderline High 130-159 (N=9097)	High 160-189 (N=3942)	Very High≥190 (N=1600)
**Martin-Hopkins**	Desirable≤70	1426 (34.3%)	0 (0.0%)	0 (0.0%)	0 (0.0%)	0 (0.0%)	0 (0.0%)
	Desirable 71-99	2736 (65.7%)	5116 (51.9%)	0 (0.0%)	0 (0.0%)	0 (0.0%)	0 (0.0%)
	Above Desirable 100-129	0 (0.0%)	4741 (48.1%)	9140 (70.4%)	0 (0.0%)	0 (0.0%)	0 (0.0%)
	Borderline High 130-159	0 (0.0%)	0 (0.0%)	3835 (29.6%)	7522 (82.7%)	0 (0.0%)	0 (0.0%)

High 160-189	0 (0.0%)	0 (0.0%)	0 (0.0%)	1575 (17.3%)	3669 (93.1%)	0 (0.0%)
Very High≥190	0 (0.0%)	0 (0.0%)	0 (0.0%)	0 (0.0%)	273 (6.9%)	1600 (100.0%)

LDL-C lipoprotein cholesterol

Supplementary Table 2 Comparison by LDL-C estimated by the Martin-Hopkins and Sampson Equations stratified by triglycerides levels, count (%)

**Triglycerides <400 mg/dL**		**Martin-Hopkins**					
	**LDL-C Value (mg/dL)**	Desirable≤70 (N=16485)	Desirable 71-99 (N=52624)	Above Desirable 100-129 (N=66507)	Borderline High 130-159 (N=40798)	High 160-189 (N=14131)	Very High≥190 (N=4175)
**Sampson**	Desirable≤70	15716 (95.3%)	1494 (2.8%)	0 (0.0%)	0 (0.0%)	0 (0.0%)	0 (0.0%)
	Desirable 71-99	769 (4.7%)	48188 (91.6%)	2473 (3.7%)	0 (0.0%)	0 (0.0%)	0 (0.0%)
	Above Desirable 100-129	0 (0.0%)	2942 (5.6%)	60848 (91.5%)	1978 (4.8%)	0 (0.0%)	0 (0.0%)
	Borderline High 130-159	0 (0.0%)	0 (0.0%)	3186 (4.8%)	37072 (90.9%)	830 (5.9%)	0 (0.0%)
	High 160-189	0 (0.0%)	0 (0.0%)	0 (0.0%)	1748 (4.3%)	12793 (90.5%)	174 (4.2%)
	Very High≥190	0 (0.0%)	0 (0.0%)	0 (0.0%)	0 (0.0%)	508 (3.6%)	4001 (95.8%)
**Triglycerides <175 mg/dL**		**Martin-Hopkins**					
	**LDL-C Value (mg/dL)**	Desirable≤70 (N=15059)	Desirable 71-99 (N=44772)	Above Desirable 100-129 (N=52626)	Borderline High 130-159 (N=29441)	High 160-189 (N=8887)	Very High≥190 (N=2302)
**Sampson**	Desirable≤70	14290 (94.9%)	521 (1.2%)	0 (0.0%)	0 (0.0%)	0 (0.0%)	0 (0.0%)
	Desirable 71-99	769 (5.1%)	41309 (92.3%)	500 (1.0%)	0 (0.0%)	0 (0.0%)	0 (0.0%)
	Above Desirable 100-129	0 (0.0%)	2942 (6.6%)	48943 (93.0%)	210 (0.7%)	0 (0.0%)	0 (0.0%)
	Borderline High 130-159	0 (0.0%)	0 (0.0%)	3183 (6.0%)	27503 (93.4%)	0 (0.0%)	0 (0.0%)
	High 160-189	0 (0.0%)	0 (0.0%)	0 (0.0%)	1728 (5.9%)	8401 (94.5%)	0 (0.0%)
	Very High≥190	0 (0.0%)	0 (0.0%)	0 (0.0%)	0 (0.0%)	486 (5.5%)	2302 (100.0%)
**Triglycerides 175<400 mg/dL**		**Martin-Hopkins**					
	**LDL-C Value (mg/dL)**	Desirable≤70 (N=1426)	Desirable 71-99 (N=7852)	Above Desirable 100-129 (N=13881)	Borderline High 130-159 (N=11357)	High 160-189 (N=5244)	Very High≥190 (N=1873)
**Sampson**	Desirable≤70	1426 (100.0%)	973 (12.4%)	0 (0.0%)	0 (0.0%)	0 (0.0%)	0 (0.0%)
	Desirable 71-99	0 (0.0%)	6879 (87.6%)	1973 (14.2%)	0 (0.0%)	0 (0.0%)	0 (0.0%)
	Above Desirable 100-129	0 (0.0%)	0 (0.0%)	11905 (85.8%)	1768 (15.6%)	0 (0.0%)	0 (0.0%)
	Borderline High 130-159	0 (0.0%)	0 (0.0%)	3 (0.0%)	9569 (84.3%)	830 (15.8%)	0 (0.0%)
	High 160-189	0 (0.0%)	0 (0.0%)	0 (0.0%)	20 (0.2%)	4392 (83.8%)	174 (9.3%)
	Very High≥190	0 (0.0%)	0 (0.0%)	0 (0.0%)	0 (0.0%)	22 (0.4%)	1699 (90.7%)

Triglycerides 400-800 mg/dL		Martin-Hopkins					
	**LDL-C Value (mg/dL)**	Desirable≤70 (N=137)	Desirable 71-99 (N=663)	Above Desirable 100-129 (N=1208)	Borderline High 130-159 (N=892)	High 160-189 (N=317)	Very High≥190 (N=229)
**Sampson**	Desirable≤70	137 (100.0%)	374 (56.4%)	2 (0.2%)	0 (0.0%)	0 (0.0%)	0 (0.0%)
	Desirable 71-99	0 (0.0%)	289 (43.6%)	681 (56.4%)	0 (0.0%)	0 (0.0%)	0 (0.0%)
	Above Desirable 100-129	0 (0.0%)	0 (0.0%)	525 (43.5%)	490 (54.9%)	0 (0.0%)	0 (0.0%)
	Borderline High 130-159	0 (0.0%)	0 (0.0%)	0 (0.0%)	402 (45.1%)	193 (60.9%)	7 (3.1%)
	High 160-189	0 (0.0%)	0 (0.0%)	0 (0.0%)	0 (0.0%)	124 (39.1%)	108 (47.2%)
	Very High≥190	0 (0.0%)	0 (0.0%)	0 (0.0%)	0 (0.0%)	0 (0.0%)	114 (49.8%)

LDL-C lipoprotein cholesterol

Supplementary Table 3 Comparison of LDL-C estimated by the Sampson and Martin-Hopkins Equations stratified by 10-Year ASCVD Risk for those participants with triglycerides < 400 mg/dL, count (%)

**ASCVD risk < 7.5**		**Sampson**					
	**LDL Value (mg/dL)**	Desirable≤70 (N=10077)	Desirable 71-99 (N=33993)	Above Desirable 100-129 (N=44397)	Borderline High 130-159 (N=25713)	High 160-189 (N=8192)	Very High≥ 190 (N=2261)
**Martin-Hopkins**	Desirable≤70	8135 (80.7%)	664 (2.0%)	0 (0.0%)	0 (0.0%)	0 (0.0%)	0 (0.0%)
	Desirable 71-99	1942 (19.3%)	29207 (85.9%)	1366 (3.1%)	0 (0.0%)	0 (0.0%)	0 (0.0%)
	Above Desirable 100-129	0 (0.0%)	4122 (12.1%)	39717 (89.5%)	951 (3.7%)	0 (0.0%)	0 (0.0%)
	Borderline High 130-159	0 (0.0%)	0 (0.0%)	3314 (7.5%)	23644 (92.0%)	392 (4.8%)	0 (0.0%)
	High 160-189	0 (0.0%)	0 (0.0%)	0 (0.0%)	1118 (4.3%)	7670 (93.6%)	94 (4.2%)
	Very High≥190	0 (0.0%)	0 (0.0%)	0 (0.0%)	0 (0.0%)	130 (1.6%)	2167 (95.8%)
**ASCVD risk 7.5 < 20%**		**Sampson**					
	**LDL Value (mg/dL)**	Desirable≤70 (N=3451)	Desirable 71-99 (N=9247)	Above Desirable 100-129 (N=11589)	Borderline High 130-159 (N=7690)	High 160-189 (N=3181)	Very High≥ 190 (N=1184)
**Martin-Hopkins**	Desirable≤70	2478 (71.8%)	79 (0.9%)	0 (0.0%)	0 (0.0%)	0 (0.0%)	0 (0.0%)
	Desirable 71-99	973 (28.2%)	7471 (80.8%)	175 (1.5%)	0 (0.0%)	0 (0.0%)	0 (0.0%)
	Above Desirable 100-129	0 (0.0%)	1697 (18.4%)	10133 (87.4%)	176 (2.3%)	0 (0.0%)	0 (0.0%)
	Borderline High 130-159	0 (0.0%)	0 (0.0%)	1281 (11.1%)	7054 (91.7%)	100 (3.1%)	0 (0.0%)
	High 160-189	0 (0.0%)	0 (0.0%)	0 (0.0%)	460 (6.0%)	2989 (94.0%)	31 (2.6%)
	Very High≥190	0 (0.0%)	0 (0.0%)	0 (0.0%)	0 (0.0%)	92 (2.9%)	1153 (97.4%)
**ASCVD risk ≥ 20%**		**Sampson**					
	**LDL Value (mg/dL)**	Desirable≤70 (N=6628)	Desirable 71-99 (N=11486)	Above Desirable 100-129 (N=8951)	Borderline High 130-159 (N=4522)	High 160-189 (N=1563)	Very High≥ 190 (N=595)
**Martin-Hopkins**	**Desirable≤70**	5007 (75.5%)	122 (1.1%)	0 (0.0%)	0 (0.0%)	0 (0.0%)	0 (0.0%)
	**Desirable 71-99**	1621 (24.5%)	9720 (84.6%)	149 (1.7%)	0 (0.0%)	0 (0.0%)	0 (0.0%)
	**Above Desirable 100-129**	0 (0.0%)	1644 (14.3%)	7962 (89.0%)	105 (2.3%)	0 (0.0%)	0 (0.0%)
	**Borderline High 130-159**	0 (0.0%)	0 (0.0%)	840 (9.4%)	4125 (91.2%)	48 (3.1%)	0 (0.0%)

**High 160-189**	0 (0.0%)	0 (0.0%)	0 (0.0%)	292 (6.5%)	1462 (93.5%)	15 (2.5%)
**Very High≥190**	0 (0.0%)	0 (0.0%)	0 (0.0%)	0 (0.0%)	53 (3.4%)	580 (97.5%)

LDL-C lipoprotein cholesterol, *ASCVD* atherosclerotic cardiovascular disease

Supplementary Fig. 1 Distribution of triglycerides in the study population. The median triglyceride level is represented by the dotted blue line.

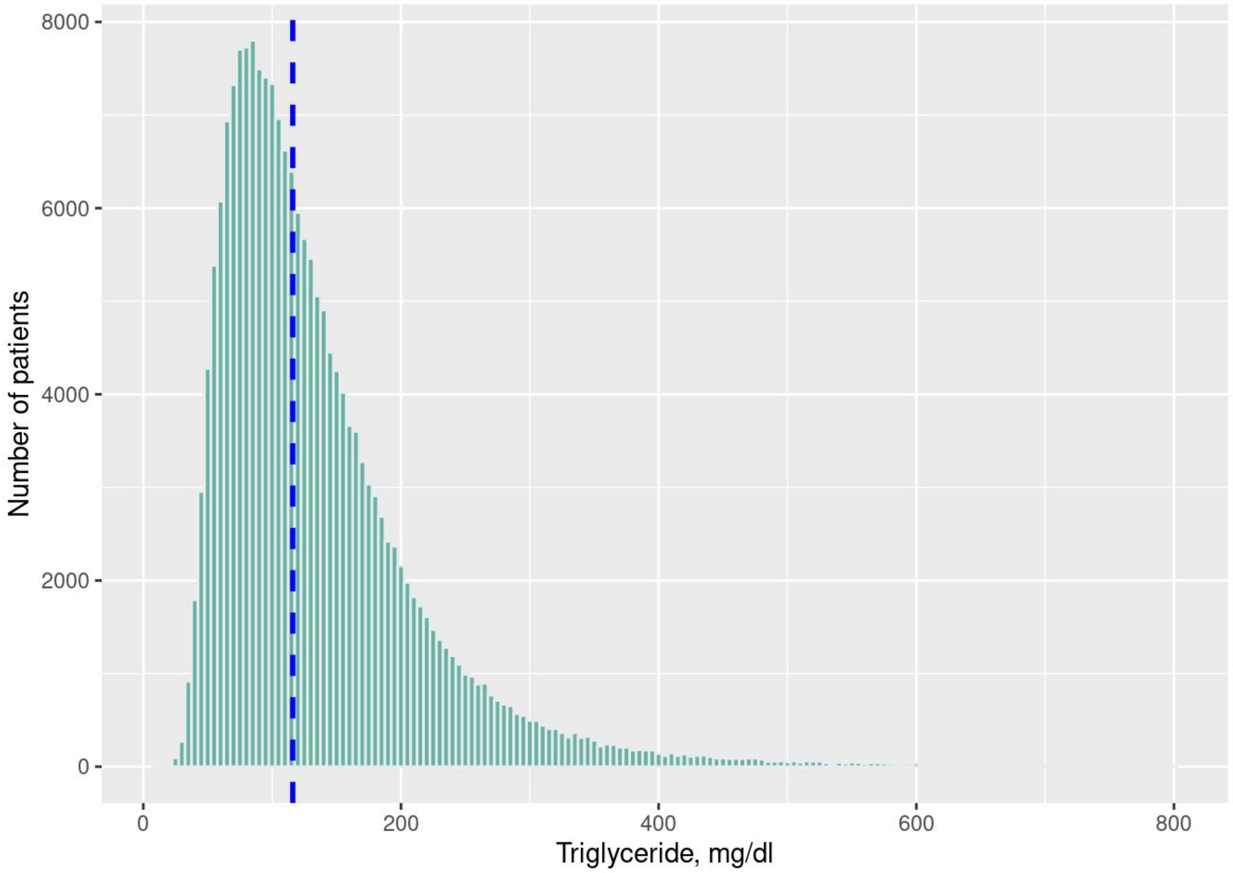

Supplementary Fig. 2 Non-HDL (mg/dL) levels by triglyceride levels for triglyceride 0-800 mg/dL.

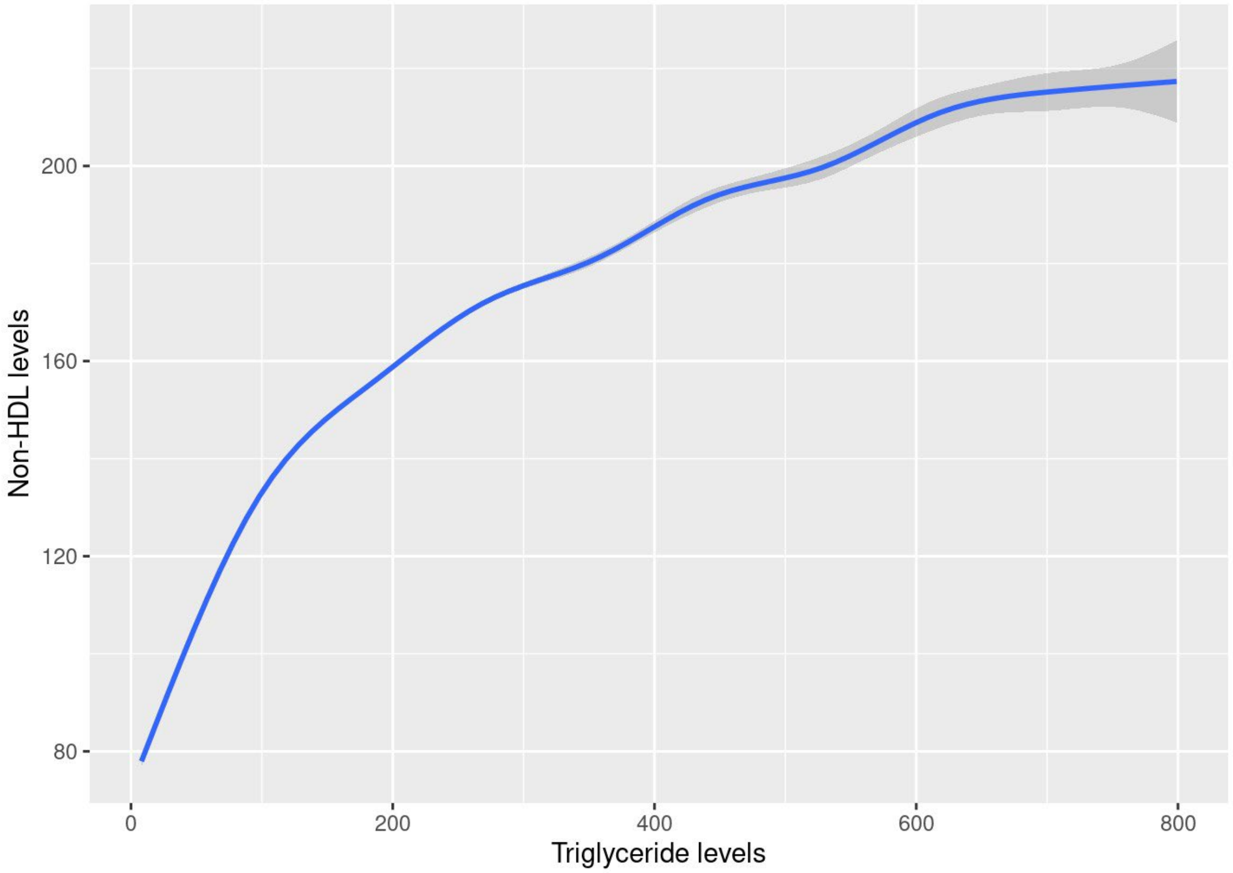

Supplementary Fig. 3 Estimated low-density lipoprotein cholesterol (LDL-C) by triglyceride levels for Martin-Hopkins and Sampson when triglyceride (400 mg/dL – 800 mg/dL).

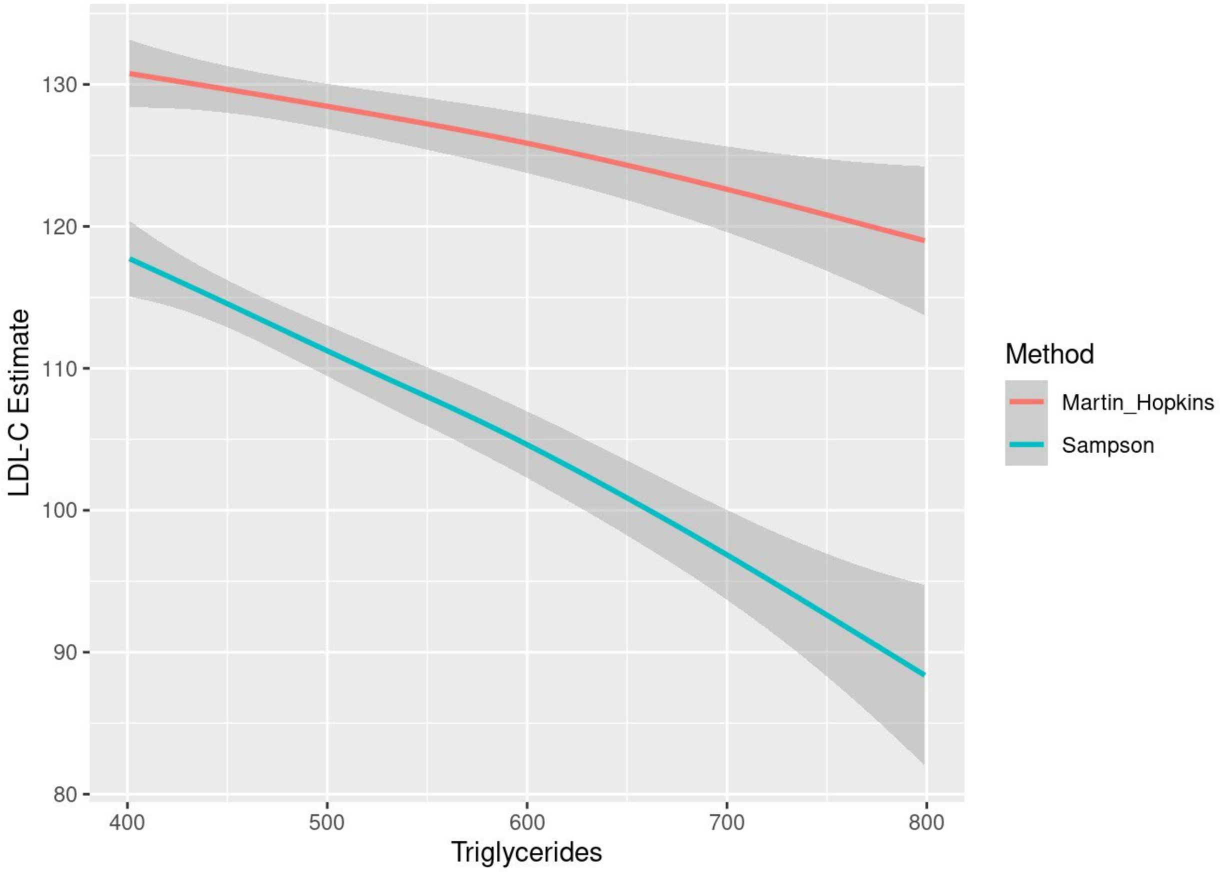

Supplementary Fig. 4 Comparison of low-density lipoprotein cholesterol (LDL-C) for diabetic patients with triglycerides < 400 mg/dL and 10-year atherosclerotic cardiovascular disease risk (ASCVD) ≥ 7.5%. (A) Concordance and discordance around LDL values of 70 mg/dL and (B) median triglyceride level for each quadrant.

A

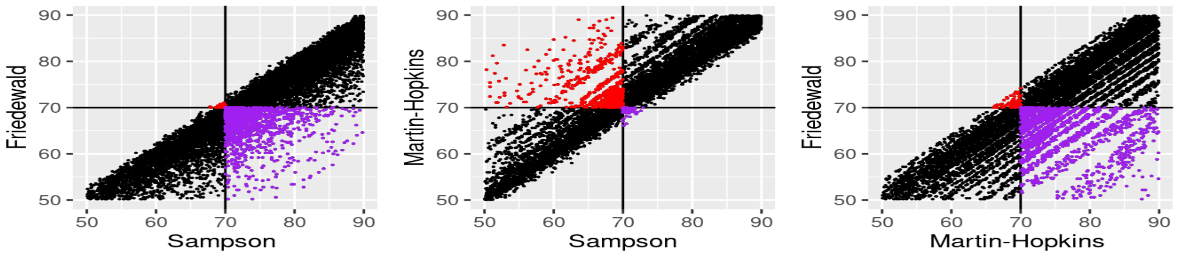

B

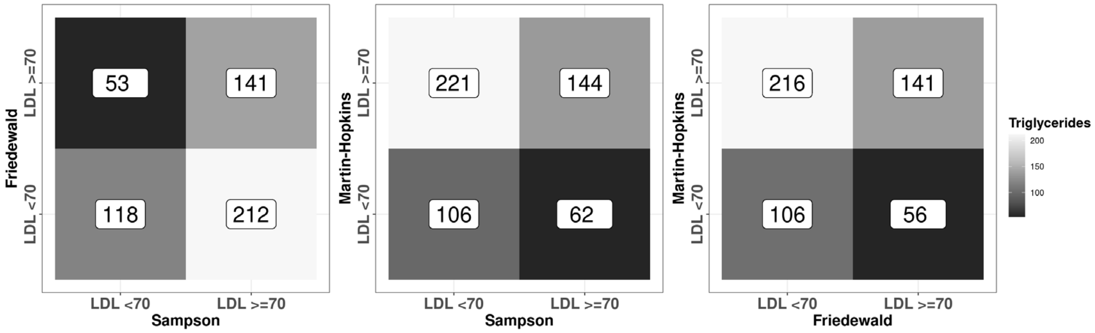

Supplementary Fig. 5 Comparison of low-density lipoprotein cholesterol (LDL-C) for diabetic patients with triglycerides < 400 mg/dL and low 10-year atherosclerotic cardiovascular disease risk (ASCVD) < 7.5%. (A) Concordance and discordance around LDL values of 100 mg/dL and (B) median triglyceride level for each quadrant.

A

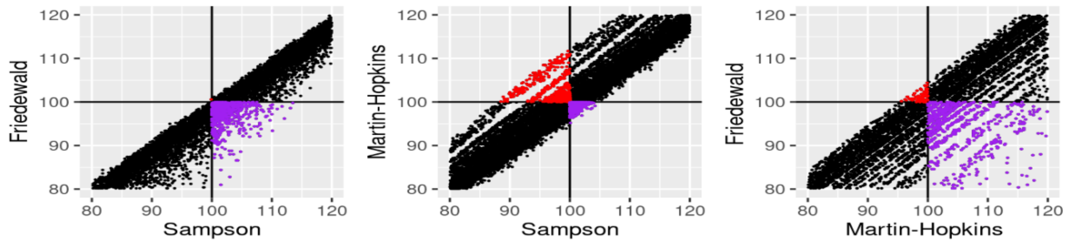

B

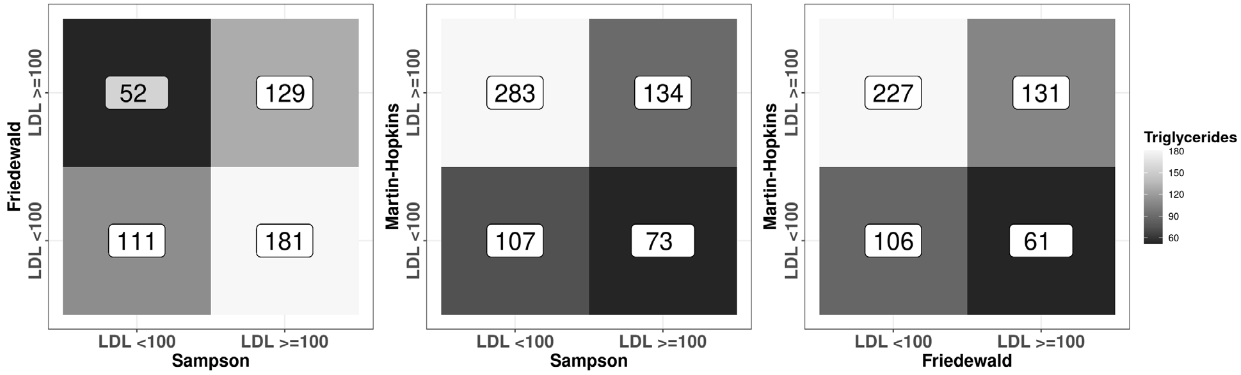

Supplementary Fig. 6 Comparison of low-density lipoprotein cholesterol (LDL-C) at the 190 mg/dL threshold by equation.

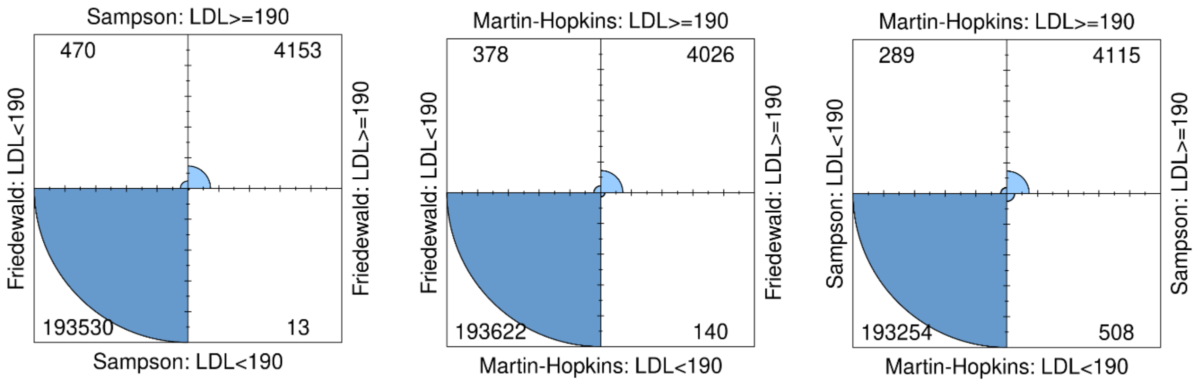

Supplement: Supplementary file 1 — Additional file 1: Table S1. Comparison of LDL-C estimated by the Friedewald and Martin-Hopkins Equations for those participants with triglycerides < 400 mg/dL and further stratified by triglycerides < 175 and 175 < 400 mg/dL, count (%). Table S2. Comparison by LDL-C estimated by the Martin-Hopkins and Sampson Equations stratified by triglycerides levels, count (%). Table S3. Comparison of LDL-C estimated by the Sampson and Martin-Hopkins Equations stratified by 10-Year ASCVD Risk for those participants with triglycerides < 400 mg/dL, count (%). Fig. S1. Distribution of triglycerides in the study population. The median triglyceride level is represented by the dotted blue line. Fig. S2. Non-HDL (mg/dL) levels by triglyceride levels for triglyceride 0-800 mg/dL. Fig. S3. Estimated low-density lipoprotein cholesterol (LDL-C) by triglyceride levels for Martin-Hopkins and Sampson when triglyceride (400 mg/dL – 800 mg/dL). Fig. S4. Comparison of low-density lipoprotein cholesterol (LDL-C) for diabetic patients with triglycerides < 400 mg/dL and 10-year atherosclerotic cardiovascular disease risk (ASCVD) ≥ 7.5%. (A) Concordance and discordance around LDL values of 70 mg/dL and (B) median triglyceride level for each quadrant. Fig. S5. Comparison of low-density lipoprotein cholesterol (LDL-C) for diabetic patients with triglycerides < 400 mg/dL and low 10-year atherosclerotic cardiovascular disease risk (ASCVD) < 7.5%. (A) Concordance and discordance around LDL values of 100 mg/dL and (B) median triglyceride level for each quadrant. Fig. S6. Comparison of low-density lipoprotein cholesterol (LDL-C) at the 190 mg/dL threshold by equation. [file 12944_2024_2188_MOESM1_ESM.pdf]
